# Biodiversity Patterns and Ecological Preferences of the Photobionts Associated With the Lichen-Forming Genus *Parmelia*

**DOI:** 10.3389/fmicb.2021.765310

**Published:** 2021-12-24

**Authors:** Patricia Moya, Arantzazu Molins, Pavel Škaloud, Pradeep K. Divakar, Salvador Chiva, Cristina Dumitru, Maria Carmen Molina, Ana Crespo, Eva Barreno

**Affiliations:** ^1^Botánica, Instituto Cavanilles de Biodiversidad y Biología Evolutiva (ICBIBE), Fac. CC. Biológicas, Universitat de València, Valencia, Spain; ^2^Department of Botany, Faculty of Science, Charles University, Prague, Czechia; ^3^Departamento de Farmacología, Farmacognosia y Botánica, Facultad de Farmacia, Universidad Complutense de Madrid, Madrid, Spain; ^4^Departamento de Biología, Geología, Física y Química Inorgánica, Escuela Superior de Ciencias Experimentales y Tecnología (ESCET), Universidad Rey Juan Carlos, Madrid, Spain

**Keywords:** distribution, habitat, microalgae, phycobiont, symbiosis, *Trebouxia*

## Abstract

The worldwide, ecologically relevant lichen-forming genus *Parmelia* currently includes 41 accepted species, of which the *Parmelia sulcata* group (PSULgp) and the *Parmelia saxatilis* group (PSAXgp) have received considerable attention over recent decades; however, phycobiont diversity is poorly known in *Parmelia* s. lat. Here, we studied the diversity of *Trebouxia* microalgae associated with 159 thalli collected from 30 locations, including nine *Parmelia* spp.: *P. barrenoae, P. encryptata, P. ernstiae, P. mayi, P. omphalodes, P. saxatilis, P. serrana, P. submontana*, and *P. sulcata*. The mycobionts were studied by carrying out phylogenetic analyses of the nrITS. Microalgae genetic diversity was examined by using both nrITS and LSU rDNA markers. To evaluate putative species boundaries, three DNA species delimitation analyses were performed on *Trebouxia* and *Parmelia*. All analyses clustered the mycobionts into two main groups: PSULgp and PSAXgp. Species delimitation identified 13 fungal and 15 algal species-level lineages. To identify patterns in specificity and selectivity, the diversity and abundance of the phycobionts were identified for each *Parmelia* species. High specificity of each *Parmelia* group for a given *Trebouxia* clade was observed; PSULgp associated only with clade I and PSAXgp with clade S. However, the degree of specificity is different within each group, since the PSAXgp mycobionts were less specific and associated with 12 *Trebouxia* spp., meanwhile those of PSULgp interacted only with three *Trebouxia* spp. Variation-partitioning analyses were conducted to detect the relative contributions of climate, geography, and symbiotic partner to phycobiont and mycobiont distribution patterns. Both analyses explained unexpectedly high portions of variability (99 and 98%) and revealed strong correlations between the fungal and algal diversity. Network analysis discriminated seven ecological clusters. Even though climatic conditions explained the largest proportion of the variation among these clusters, they seemed to show indifference relative to climatic parameters. However, the cluster formed by *P. saxatilis* A/*P. saxatilis* B/*Trebouxia* sp. 2/*Trebouxia* sp. S02/*Trebouxia* sp. 3A was identified to prefer cold-temperate as well as humid summer environments.

## Introduction

Lichens are complex systems individualized from cyclical symbiotic associations (Khakhina et al., 1993) which represent the best example of symbiotic associations between organisms belonging to several taxonomically and phylogenetically distinct groups. Lichens are composed of a fungus, or mycobiont, and an internal population of photosynthetic organisms, or photobionts, which may be green microalgae (phycobionts) and/or cyanobacteria (cyanobionts) (Hawksworth and Honegger, 1994). Many lichen-forming fungal species are widely distributed and occupy ca. 8% of the surface of the planet (Nash, 2008). However, phycobiont distribution patterns seem to show their own ecological preferences, independently of the lichen forming fungus (Peksa and Škaloud, 2011; Řídká et al., 2014; Rolshausen et al., 2020; Moya et al., 2021). In fact, both restricted and globally distributed phycobionts have been recorded (Muggia et al., 2014; Vančurová et al., 2018; Garrido-Benavent et al., 2021).

Success in the development of the lichen thallus across micro and macro ecological scales is clearly influenced by the presence of the two main symbionts in the environment, and by the degree of selectivity and specificity that either symbiont shows for each other (Beck et al., 2002; Yahr et al., 2004, 2006). Terms such as specificity and selectivity have been defined by several authors (Muggia and Grube, 2018); however, these concepts are mainly determined by sampling bias and should be constantly revised. In general, mycobionts which show a high specificity for algal partners tend to accept only single algal lineages (Piercey-Normore and DePriest, 2001; O’Brien et al., 2013; Leavitt et al., 2015; Magain et al., 2017; Kosecka et al., 2020; Mark et al., 2020; Pino-Bodas and Stenroos, 2020; Garrido-Benavent et al., 2021), while mycobionts which are generalist can associate with many different algal lineages (Wirtz et al., 2003; Muggia et al., 2013; Sadowska-Deś et al., 2014); this has also been reported for photobionts and their preference toward fungal partners (Peksa and Škaloud, 2011). Specific or generalist associations among lichen symbionts are not random, but rather are modulated by ecological, environmental and evolutionary factors (Beck et al., 2002; Miadlikowska et al., 2006; Leavitt et al., 2015; Grube and Wedin, 2016; Chagnon et al., 2019), which have significant impacts on the structure of lichen communities and species distribution (Muggia et al., 2014; Sork and Werth, 2014; Steinová et al., 2019). Many analyses have pointed out that climatic conditions are the most important factor shaping phycobiont distribution patterns (Kroken and Taylor, 2000; Helms et al., 2003; Fernández-Mendoza et al., 2011; Peksa and Škaloud, 2011; Řídká et al., 2014); however, the influence of factors such as phylogenetic and evolutionary specialization, reproductive strategy, the presence of compatible phycobionts as well as ecological factors should not be overlooked (Yahr et al., 2006; Muggia et al., 2013; Printzen et al., 2013; Singh et al., 2017; Ertz et al., 2018; Pardo-De la Hoz et al., 2018; Molins et al., 2021).

*Trebouxia* Puymaly Tschermak-Woess (Trebouxiales, Trebouxiaceae) is one of the most frequent lichen symbionts, associating with over 7,000 species of lichen-forming fungi from across a wide range of fungal classes (Lücking et al., 2017). *Trebouxia* is among the most widespread phycobionts, and it associates with a broad range of lichen-forming fungi; estimated to be 80% in temperate regions and more than 20% worldwide (Muggia et al., 2020; Nelsen, 2021). To date, 29 *Trebouxia* species have been described based on a combination of morphological traits and genetic diversity (Muggia et al., 2018). More recently, four major clades have been confirmed within *Trebouxia* in a multi-locus phylogeny (Muggia et al., 2020). These includes clades A (arboricola/gigantea-type), C (corticola-type), I (impressa/gelatinosa-type), and S (simplex/jamesii-type). Some *Trebouxia* algae of clade S, specifically associating with the lichen-forming fungus *Cetrariella delisei*, were provisionally segregated into a new clade, D (Xu et al., 2020).

The ecologically relevant lichen-forming genus *Parmelia* has received considerable attention over recent decades. This genus is widely distributed in cold-temperate Europe, North America, and eastern Asia (Hale, 1987; Hawksworth et al., 2008, 2011; Crespo et al., 2010). The genus *Parmelia* s. str. includes 41 currently accepted species which belong to the parmelioid crown of Parmeliaceae. Within *Parmelia* s. str., the *Parmelia sulcata* group (PSULgp) includes sorediate species; 6 spp and the *Parmelia saxatilis* group (PSAXgp) mainly isidiate species; 12 spp (Divakar et al., 2016; Molina et al., 2017). Both isidia and soredia are dispersal packages of both fungus and algae (vegetative reproduction). Several molecular studies on PSAXgp and PSULgp have been published and have revealed the presence of cryptic species (Crespo et al., 1997, 1999, 2002). Over the last two decades’ molecular investigations focusing on PSAXgp and PSULgp from the Iberian Peninsula have been carried out. These studies allowed us to described *Parmelia serrana* (Molina et al., 2004) and *Parmelia encryptata* (Molina et al., 2011a). Also, Divakar et al. (2005) detected additional *Parmelia barrenoae* species in the PSULgp from the Iberian Peninsula, North America and Africa. More recently, *Parmelia rojoi* was segregated from PSAXgp from Spain (Crespo et al., 2020). Apart from these, *Parmelia imbricaria, Parmelia mayi*, and *Parmelia sulymae* were segregated from PSAXgp in North America (Molina et al., 2011b,^2017^). The species delimitation in *Parmelia discordans/Parmelia omphalodes*, and *Parmelia hygrophila/Parmelia submontana* complexes remains unclear (Molina et al., 2017; Ossowska et al., 2019).

Contrasting patterns of worldwide distributions in some *Parmelia* spp., and geographically restricted distributions in other species of this genus, have been documented (Molina et al., 2011a; Crespo et al., 2020). However, photobiont diversity associated with these lichen-forming fungal species remains almost unknown. Leavitt et al. (2015) analyzed the algal nrITS region from 11 mycobiont genera of Parmeliaceae and Lecanoreceae (but they did not include *Parmelia*). They explored the interactions patterns in these species based on habitat preferences and mycobiont phylogeny, and concluded that: “fungal specificity and selectivity for algal partners played a major role in determining lichen partnerships.” Recently, Ossowska et al. (2019) evaluated the genetic variability of phycobionts within the *Parmelia omphalodes* group, which include *P. omphalodes*, *P. discordans*, and *P. pinnatifida*. All taxa investigated in this study showed moderate selectivity in their phycobiont choice and associated only with *Trebouxia* from clade S. Dumitru (2019) performed a preliminary study of phycobiont diversity in *Parmelia* spp. distributed in the Iberian Peninsula. The results of this study were the starting point of the present analyses. Furthermore, we do not know the distribution pattern of phycobiont species associated with the remaining *Parmelia* lichen taxa, and this may play a crucial role in speciation of this lichen forming fungal species.

Thus, as ecological requirements of phycobionts could be directly related with the host distribution, in the present study we focused on *Trebouxia* biodiversity in the lichen-forming genus *Parmelia* to better understand their global spatial distribution and ecological preferences.

## Materials and Methods

### Lichens Sampled

In this study 159 thalli were sampled from 30 locations (Supplementary Table 1) including nine formally described *Parmelia* spp., viz. *Parmelia barrenoae*, *Parmelia encryptata*, *Parmelia ernstiae, Parmelia mayi, Parmelia omphalodes, Parmelia saxatilis, Parmelia serrana, Parmelia submontana*, and *Parmelia sulcata*. Both fresh (*n* = 133) and herbarium samples (*n* = 26) were included in the analyses. Fresh specimens were stored at −20°C after sampling.

### DNA Extraction, PCR Amplification and Sanger Sequencing

Lichen thalli were processed as described in Moya et al. (2021), in brief: specimens were examined under a stereo microscope to remove surface contamination (mainly tree bark). Selected fragments from different parts of each thallus were randomly excised and pooled together. These fragments were superficially sterilized following Arnold et al. (2009).

Total genomic DNA was isolated and purified using the DNeasy Plant Mini kit (Qiagen, Hilden, 121 Germany) following the manufacturer’s instructions. The mycobionts and the phycobionts were identified by Sanger sequencing. The fungal nuclear ribosomal internal transcribed spacer (nrITS) was amplified using the primer pair ITS1F (Gardes and Bruns, 1993) and ITS4 (White et al., 1990). Two algal loci were amplified to confirm the identity of the main phycobiont in the 159 lichen thalli; a region of the chloroplast LSU rDNA gene, using the algal specific primers 23SU1 and 23SU2 (del Campo et al., 2010) and the nrITS using the primer pair nr-SSU-1780 (Piercey-Normore and DePriest, 2001) and ITS4 (White et al., 1990).

All PCR reactions were performed following Moya et al. (2018). PCR products were visualized on 2% agarose gels and purified using the Gel Band Purification Kit (GE Healthcare Life Science, Buckinghamshire, United Kingdom). Amplified PCR products were sequenced with an ABI 3730XL using the BigDye Terminator 3.1 Cycle Sequencing Kit (Applied Biosystems, Foster City, CA, United States). Sanger sequences were visualized and manually evaluated with Chromas v 2.6.6.0.^1^ Taxonomic assignment of Sanger sequenced strains was checked by conducting phylogenetic analyses.

### Mycobiont Phylogenetic Analyses

Fungal sequence identity was evaluated using the mega-BLAST search function in GenBank (Sayers et al., 2011). Newly obtained data set (*n* = 159) were aligned with an nrITS dataset published in our previous DNA barcoding study (Divakar et al., 2016). A multiple sequence alignment was performed using the MAFFT v 7.0 program (Katoh and Toh, 2008) implementing the G-INS-I alignment algorithm, “1PAM/K = 2” scoring matrix, with an offset value of 0.0, and the remaining parameters set to default values. Phylogenetic analysis was carried out using the maximum likelihood (ML) approach. The ML analysis was performed using the RAxML v 8.2.6 program (Stamatakis, 2014) as implemented on the CIPRES Web Portal with the GTRGAMMA substitution model. Nodal support was evaluated using the “rapid bootstrapping” option with 1,000 replicates (Stamatakis et al., 2008). Only clades that received bootstrap support of greater than, or equal to, 70% (Hillis and Bull, 1993) were considered as well supported. The phylogenetic tree was drawn in FigTree v 1.4.1 (Rambaut, 2012).

### Phycobiont Phylogenetic Analyses

*Trebouxia* phycobiont identity was further checked by phylogenetic assignment. For the nrITS, a multiple alignment was constructed including the *Trebouxia* data set obtained by Sanger sequencing (*n* = 159) and 10 selected sequences from clades S and I (Muggia et al., 2020). *Asterochloris mediterranea* (KP257398) was included as an outgroup. A multiple alignment was built using MAFFT v 7.0 (Katoh et al., 2002; Katoh and Toh, 2008) using default parameters. The substitution model TVM + G was the most accurate for the nrITS region according to the Akaike information criterion (AIC) using JModelTest v 2.1.4 (Darriba et al., 2012). The phylogenetic trees were inferred by Bayesian inference (BI) and maximum likelihood (ML) approaches. ML analysis was implemented in RAxML v 8.1.11 (Stamatakis, 2006) using the GTRGAMMA substitution model. Bootstrap support (BS) was calculated based on 1,000 pseudoreplicates (Stamatakis et al., 2008). BI was carried out in MrBAYES v 3.2 (Ronquist et al., 2012). Settings included two parallel runs with six chains over 20 million generations, starting with a random tree and sampling after every 200th step. We discarded the first 25% of data as burn-in, and the corresponding posterior probabilities (PPs) were calculated from the remaining trees. Estimated sampled sized (ESS) values above 200, and potential scale reduction factor values approaching 1,000, were considered indicators of chain convergence. All analyses were performed with the CIPRES Science Gateway v 3.3 (Miller et al., 2011). Phylogenetic trees were visualized in FigTree v 1.4.1 (Rambaut, 2012).

In the case of the chloroplast genome marker, a multiple alignment was built including the *Trebouxia* obtained (*n* = 129) and a selection of *Trebouxia* type species belonging to clades S and I available from the Culture Collection of Algae at Göttingen University (SAG) and from the Culture Collection of Algae at the University of Texas (UTEX). *Asterochloris mediterranea* (KP257332) was included as an outgroup. The alignment and phylogenetic analysis were carried out as previously described for the nrITS. The best-fit substitution model for this region was GTR + I + G.

### Delimitation of Mycobiont and Phycobiont Species

To estimate putative species boundaries in the concatenated *Trebouxia* (nrITS + LSU rDNA phycobiont) and *Parmelia* (nrITS mycobiont) data sets, we performed three species delimitation analyses (GMYC, bPTP, ABGD). All analyses were performed following Vančurová et al. (2018). Summarizing, the Bayesian analyses with BEAST v 1.8.2 were performed (Drummond et al., 2012) to obtain ultrametric trees under the assumption of uncorrelated log normal relaxed molecular clocks. For each of the alignment partitions, the most appropriate substitution model was estimated using the Bayesian information criterion (BIC) as implemented in JModelTest2 (Guindon and Gascuel, 2003; Darriba et al., 2012). The models were selected as follows: GTR + G + I for both algal and fungal ITS1 and ITS2 partitions, HKY for both algal and fungal 5.8 rDNA partitions, and GTR + G + I for the LSU rDNA partition. The analyses were performed under the constant population size coalescent, as the tree prior and Ucld mean prior were set to exponential distribution with mean 10 and initial value 1. Five MCMC analyses were run for 30 million generations, sampling every 10,000 generations. The outputs were diagnosed for convergence using Tracer v. 1.7 (Rambaut et al., 2018), and the five tree files were merged using the burn−in set to 3 million generations (all ESS values of the merged data set were above 900). A consensus tree was generated using TreeAnnotator v 1.8.2.^2^ The GMYC analysis was performed on the ultrametric consensus tree under the single−threshold model, using the SPLITS package (Monaghan et al., 2009) in R v 4.0.5 (R Core Team, 2013). The bPTP analysis was also performed on the ultrametric consensus tree, using the bPTP web Server^3^. The analysis was run for 200,000 generations, using 0.3 burn−in and 100 thinning. Both ML and Bayesian solutions were examined. Finally, the ABGD analysis was performed on the concatenated algal and ITS rDNA fungal alignments, using the ABGD web server^4^. Genetic distances were calculated using the K80 model, and the model parameters were set to P_min_ 0.001, P_max_ 0.01, Steps 10 and Nb bins 20. Separate analyses were run under varying relative gap width values (X set to 0.1, 0.3, 0.5, 0.8, 1.0) to assess the consistency of the inferred groups. The final OTU delimitation was selected as a consensus between all three delimitation approaches described above. To assign names to each recovered lineage of both *Trebouxia* and *Parmelia*, we used either the names of previously described species or the clade names of yet undescribed lineages, following the labeling system of previous studies.

### Mycobiont and Phycobiont Interaction Patterns

To identify patterns in specificity and selectivity, the diversity and abundance of phycobionts were identified for each *Parmelia* species. A mycobiont-phycobiont interaction network was implemented, where *Trebouxia* and *Parmelia* were grouped at species level. Bipartite networks were constructed using the bipartite package in R (R Core Team, 2013). The topological properties of the network were determined by modularity analyses using Gephi v 0.9.2 (Bastian et al., 2009). The default algorithms were used and the analysis was performed under the following settings: undirected graph type, force atlas with 0.1 inertia, repulsion 4,000, strength, attraction strength 5, and maximum displacement 1. We applied the auto stabilize function with strength 100, sensibility 0.2, and gravity 50. To improve readability and esthetics, a no-overlap algorithm to spread nodes apart was used. The weighted average degree was calculated, and this criterion was applied to adjust the nodes by sizes. The modularity analysis was performed using the Louvain method and the modules were visualized by colors.

### Variation Partitioning

Two variation partitioning analyses were performed following Vančurová et al. (2018). Summarizing, first the relative effects of geography, climate, substrate chemistry and the symbiotic partner on the variance in photobiont as well as mycobiont diversity were evaluated. Second, we tested whether the modules identified by the modularity analyses (see above) can be differentiated by geography, climate and substrate chemistry. From the entire data set of 159 specimens, 14 were excluded due to insufficient substrate/habitat data, resulting in a data set of 145 samples. The relative effects of climate, substrate/habitat, geographical distance and the symbiotic partner on the variance in photobiont as well as mycobiont diversity were analyzed by variation partitioning in redundancy analysis, using the varpart function in the vegan package (Oksanen et al., 2017). The phylogenetic distances of phycobionts, or mycobionts, were used as a response variable, coded as the first 10 PCoA axes. Climatic data were obtained from the CHELSA Bioclim database (Karger et al., 2017) at a resolution of 2.5 arc minutes. At each sampling site, climatic data were obtained by applying a 5 km buffer to limit the effects of spatial bias. Geographical distance values (latitude and longitude) were transformed to the principal coordinates of neighbor matrices (PCNM) vectors representing the geographical distances at various spatial scales (Borcard et al., 2004). PCNM vectors were calculated based on the pairwise geographical distances obtained by the distGPS function in the BoSSA package (Lefeuvre, 2018). The first 100 PCNM were used for the analysis. All response variables (19 environmental variables, 5 substrates, 8 PCNM scores) were condensed into principal component variables (PCs). The Broken−stick distribution (Jackson, 1993) was used to select which principal components to include in variation partitioning analysis, using the bstick function in the vegan package (Oksanen et al., 2017). Accordingly, the first three PCA axes were selected in all response variables, with the exception of geography where only two axes were retained. To detect and visualize the differential ordination (tendency or strategies) of samples in the hyperspace, we performed Principal component analyses (PCA) according to climatic factors (BIO1–BIO19) and the grouped results depending on the distribution patterns of lichen species and the genus of the mycobiont host. The differences were visualized by box plots. The significance of a difference between groups was tested by Wilcox tests using the ggsignif package (Ahlmann-Eltze and Patil, 2021). All analyses were performed in R v 4.0.5 (R Core Team, 2013), using RStudio v 1.2.133583.

## Results

### Phylogenetic Analyses and Delimitation of Species

#### Mycobiont

**FIGURE 1:**
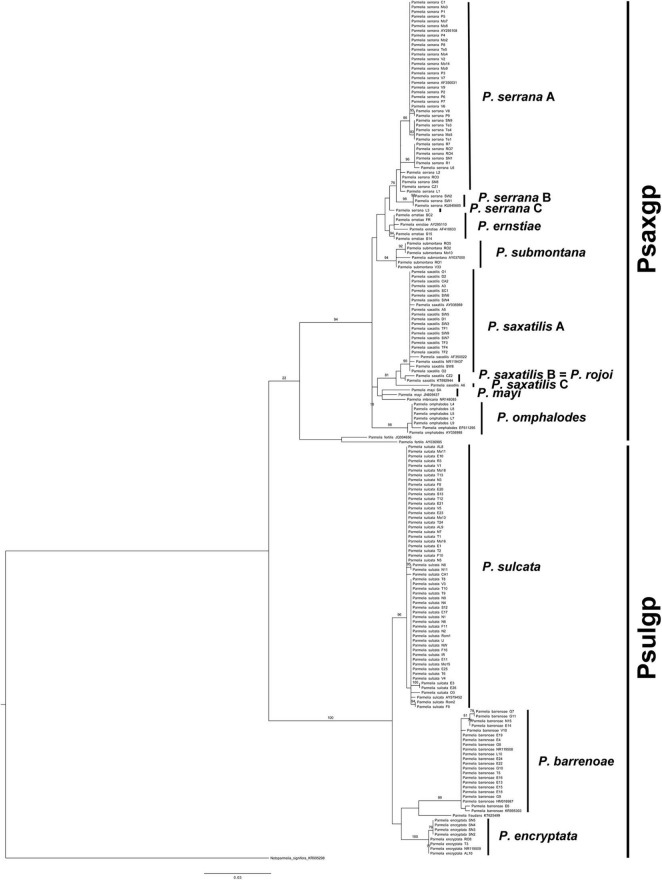
Maximum likelihood (ML) internal transcribed spacer (nrITS) tree of the *Parmelia* specimens. (ML) bootstrap values ≥ 70% from the RAxML analysis are indicated above branches.

The tree topology obtained (Figure 1) was fully congruent with previously described *Parmelia* phylogenies (Molina et al., 2017). The phylogeny showed three major clades: *P. fertilis*, PSULgp and PSAXgp. PSAXgp included seven clades, viz. *P. ernstiae, P. imbricaria, P. mayi, P. omphalodes*, *P. saxatilis, P. serrana*, and *P. submontana*. PSULgp included four clades: *P. sulcata* s. str., *P. barrenoae*, *P. encryptata*, and *P. fraudans*.

To evaluate putative species boundaries, three species delimitation analyses (GMYC, bPTP, and ABGD) were performed in the *Parmelia* data set (*n* = 159). These analyses delimited 13 species clusters (Supplementary Figure 1), viz. *P. sulcata*, *P. encryptata*, *P. barrenoae*, *P. serrana* A, *P. serrana* B, *P. serrana* C, *P. ernstiae, P. submontana, P. omphalodes, P. saxatilis* A, *P. saxatilis* B = *P. rojoi, P. saxatilis C*, and *P. mayi.* Ten of the lineages could be placed in formally described species, whereas *P. serrana* B (samples SW1, SW2 from Sweden and KU845685 from Poland), *P. serrana* C (specimen L3 from Ávila, Spain), and *P. saxatilis* C (specimen A6 from Lleida, Spain) are undescribed lineages in *Parmelia*.

#### Phycobiont

**FIGURE 2:**
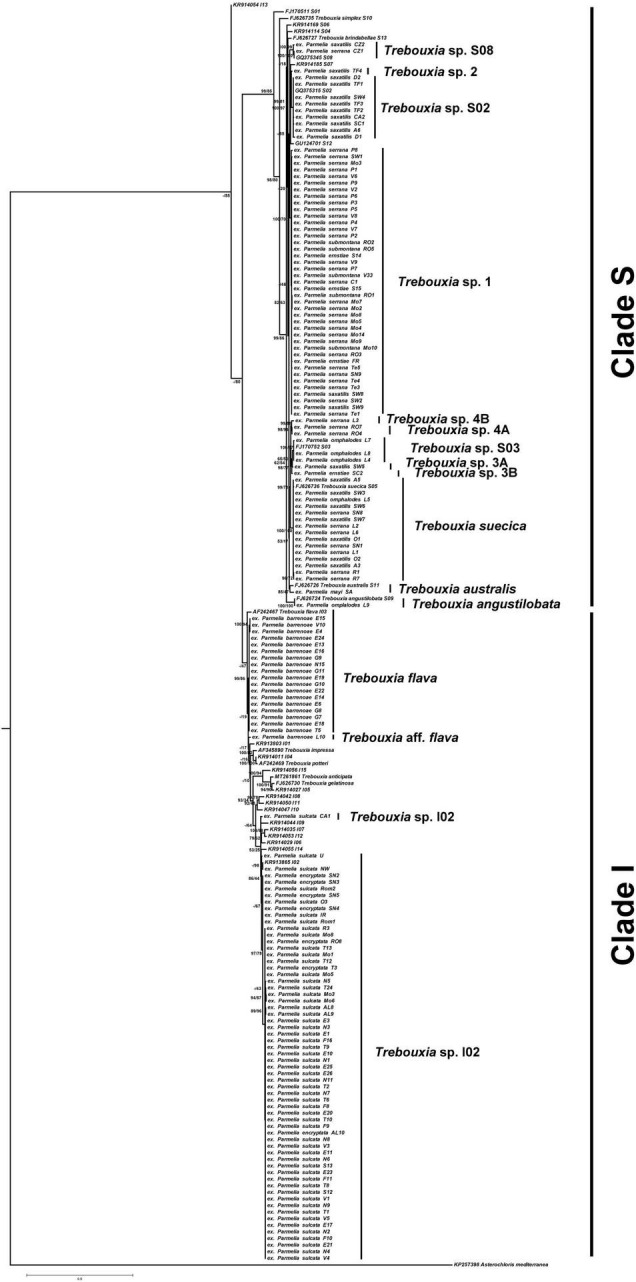
*Trebouxia* phylogenetic analysis. Rooted nrITS gene tree representing 192 *Trebouxia* sequences, including 10 well–accepted *Trebouxia* species from SAG and UTEX retrieved from the GenBank. Newly generated sequences are marked as ex *Parmelia* spp._locality-code_number. Fifteen *Trebouxia* species detected were indicated. Values at nodes indicate statistical support estimated by two methods: bootstrap support (BS, RAxML analysis) and posterior probabilities (PP, MrBayes analysis). Scale bar shows the estimated number of substitutions per site.

A total of 15 *Trebouxia* microalgae species were detected in the 159 specimens analyzed (Figure 2). As mentioned in the introduction, *Trebouxia* spp. tended to form five well-supported monophyletic clades: A, C, I, S, and D. According to this accepted *Trebouxia* clade code (Leavitt et al., 2015; Moya et al., 2017, 2021; Muggia et al., 2020; Xu et al., 2020), all sequences generated in this study belong exclusively to clades S and I. LSU rDNA phylogeny corroborated the *Trebouxia* assignment at clade level (S and I), but showed less resolution at a species level (Supplementary Figure 2).

To estimate putative species boundaries GMYC, bPTP, and ABGD species delimitation analyses were performed on the *Trebouxia* concatenated nrITS and LSU rDNA data set (*n* = 159). These three analyses delimited 15 species clusters (Supplementary Figure 3), viz. *Trebouxia* sp. I02, *T. flava*, *Trebouxia* aff. f*lava*, *Trebouxia* sp. 1, *Trebouxia* sp. S08, *Trebouxia* sp. S02, *Trebouxia* sp. 2, *T. suecia*, *Trebouxia* sp. S03, *Trebouxia* sp. 3A and 3B, *Trebouxia* sp. 4A and B, *T. australis* and *T. angustilobata*. Eight of the lineages could be placed in formally *Trebouxia* described species, whereas *Trebouxia* aff. f*lava* (ex *P. barrenoae* from Ávila, Spain), *Trebouxia* sp. 1 (ex *P. saxatilis* A, *P. serrana* A and B, *P. submontana*, and *P. ernstiae*), *Trebouxia* sp. 2 (ex *P. saxatilis* A from Chile), *Trebouxia* sp. 3A (ex *P. saxatilis* A from Sweden) and B (ex *P. ernstiae* from Scotland), *Trebouxia* sp. 4A (ex *P. serrana* A from Ronda, Spain) and B (ex *P. serrana* C from Ávila, Spain) were newly detected.

#### The Associations Among Phycobiont, Mycobiont, and Environmental Conditions

**FIGURE 3:**
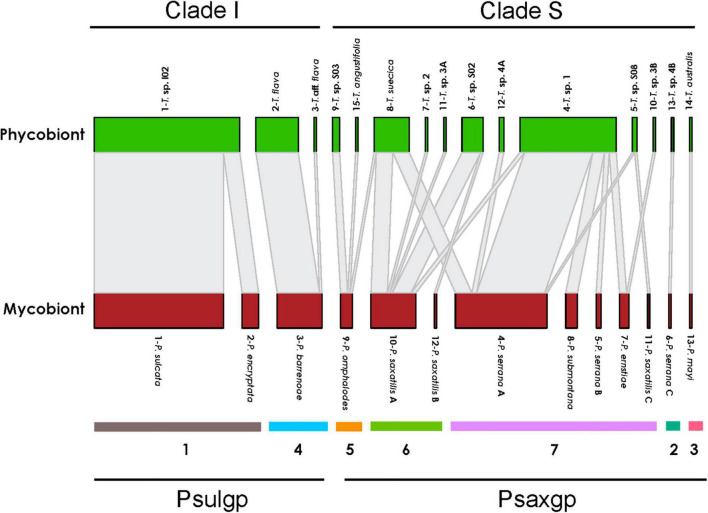
Interaction network structure between lichen mycobiont species-level lineages in the genus *Parmelia* and phycobiont species-level lineages. The width of the links is proportional to the number of specimens forming the association. Seven modules determined by modularity analysis were indicated. PSAXgp and PSULgp mycobionts and *Trebouxia* Clade I and Clade S are highlighted.

Patterns in specificity and selectivity were identified for each *Parmelia* species (Figure 3). High specificity of each *Parmelia* group for a given *Trebouxia* clade level was observed; PSULgp (*P. sulcata*, *P. barrenoae*, *P. encryptata*) associated only with clade I, and PSAXgp (*P. serrana* A, *P. serrana* B, *P. serrana* C, *P. ernstiae, P. submontana, P. omphalodes, P. saxatilis A, P. saxatilis B* = *P. rojoi, P. saxatilis C*, and *P. mayi*) with clade S. However, the degree of specificity is different within each group, since the PSAXgp mycobionts were less specific and associate with 12 *Trebouxia* spp. (*Trebouxia* sp. 1, *Trebouxia* sp. S08, *Trebouxia* sp. S02, *Trebouxia* sp. 2, *T. suecia*, *Trebouxia* sp. S03, *Trebouxia* sp. 3A and 3B, *Trebouxia* sp. 4A and 4B, *T. australis* and *T. angustilobata*), meanwhile those of PSULgp interacted with only three *Trebouxia* spp. (*Trebouxia* sp. I02, *T. flava* and *Trebouxia* aff. *flava*). Twelve *Trebouxia* lineages showed specificity toward a single *Parmelia* spp. (i.e., *T*. *australis*-*P. mayi* or *Trebouxia* sp. 4B-*P. serrana* C). Others were not specific toward a single mycobiont, but associated mainly with one fungal species-level lineage. For example, *Trebouxia* sp. 1 accepts five fungal species-level lineages, but prefers *P. serrana*.

**FIGURE 4:**
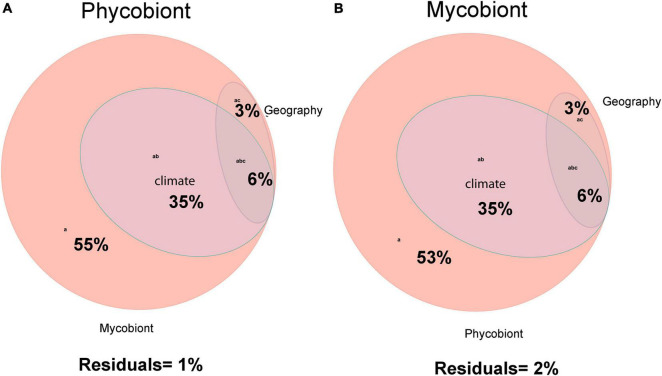
**(A)** Venn’s diagram showing the variation in distribution of phycobionts associated with the lichen-forming fungal genus *Parmelia* explained by effects of climate, geography and the mycobiont. **(B)** Venn’s diagram showing the variation in distribution of *Parmelia* mycobionts associated with the lichen-forming fungal genus *Parmelia* explained by effects of climate, geography and the phycobiont.

Variation-partitioning analyses were performed to detect the relative contributions of climate, geography and symbiotic partner to phycobiont and mycobiont distribution patterns (Figure 4). Both analyses explained unexpectedly high portions of the variability.

Among the phycobionts, climatic conditions, geography and the mycobiont explained 99% of the variation (Figure 4A). The largest proportion of the variation was explained by the symbiotic partner (55% independent effect and 99% in combination with other variables). Climatic conditions explained 42% of the variability, but explained nothing independently. The variables associated with geography explained 9% of the variability shared with other variables.

In the phylogeny of the mycobionts, climatic conditions, geography and the phycobiont explained 98% of the variation (Figure 4B). The greatest proportion of the variation (53% independent effect and 98% in combination with other variables) was explained by the symbiotic partner. The same proportions as in the phycobionts were detected for the climatic conditions and geography.

#### Correlation Between Bioclimatic Variables and Symbiont Association Pattern

Network analysis has been applied to investigate the topological properties of the mycobiont-phycobiont interactions. Our network analysis discriminated seven ecological clusters (Supplementary Figure 4A and listed below), four of them including 96% of all taxa. These four main ecological clusters included 62 (Cluster 1), 11 (Cluster 4), 17 (Cluster 6), and 31 (Cluster 7) taxa. The remaining three clusters comprised only a single taxon (Clusters 2 and 3) and three taxa (Cluster 5), respectively.

1—*P. sulcata*/*P. encryptata*/*Trebouxia* sp. I02
2—*P. serrana* C/*Trebouxia* sp. 4B
3—*P. mayi*/*T. australis*
4—*P. barrenoae*/*T. flava*/*Trebouxia* aff. *flava*
5—*P. omphalodes*/*T. angustilobata*/*Trebouxia* sp. S03
6—*P. saxatilis* A/*P. saxatilis* B/*Trebouxia* sp. 2/*Trebouxia* sp. S02/*Trebouxia* sp. 3A
7—*P. serrana* A/*P. saxatilis* C/*P. submontana*/*P. serrana* B/*P. ernstiae*/*Trebouxia* sp. S08/*Trebouxia* sp. 4A/*Trebouxia* sp. 1/*Trebouxia* sp. 3B

To identify the factors that shape these seven identified clusters, we performed a second variation-partitioning analysis (Supplementary Figure 4B). We analyzed the relative contributions of climate, geography and substrate to the module associations. The response variables explained 58% of the total variation. The largest proportion of the variation was elucidated by the climatic conditions (27% independent effect and 39% in combination with other variables). The second most important variable was the substrate, which explained 13% of the variability independently, despite 7% being shared with other variables. The third variable, geography, explained only 6% of the variation (independent effect, 7% in combination with other variables).

**FIGURE 5:**
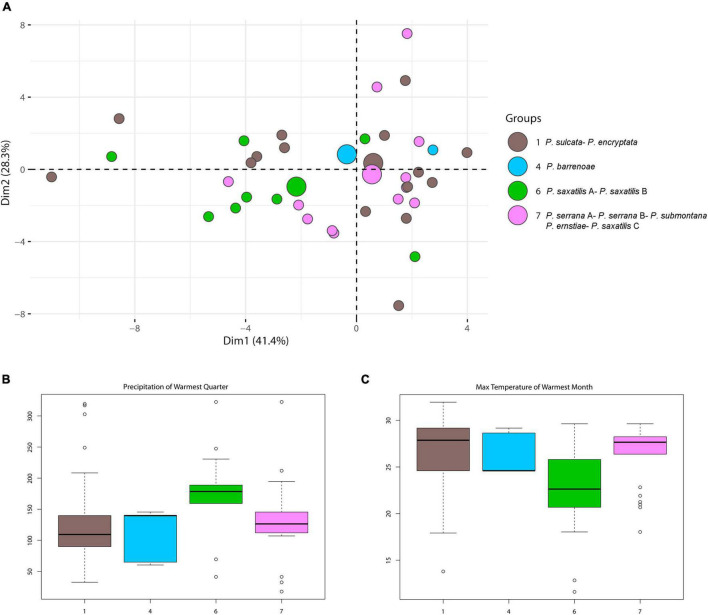
**(A)** PCA result for modules 1, 4, 6, and 7 distributions depending on climatic factors. Large circles represent group centroids. **(B,C)** Box-plot diagram representing differences in climate preferences in selected modules 1, 4, 6, and 7. Climatic data were obtained from the Global Climate Data – WorldClim. **(B)** BIO5 = Max temperature of warmest month (°C) and **(C)** BIO18 = precipitation of warmest quarter (°C).

PCA analyses were performed to identify which clusters were represented as a function of climate (Supplementary Figure 5). PCA ordination of all analyzed species resulted in a 69.5% cumulative variance, explained on the first 2 axes (first-40.8%, second-28.7%). Even though climatic conditions explained the largest proportion of the variation among these clusters, they seemed to show indifference relative to climatic parameters (Supplementary Figure 5B). However, slight differences among the groups were observed when only the major clusters were analyzed by PCA, with Cluster 6 (*P. saxatilis* A/*P. saxatilis* B/*Trebouxia* sp. 2/*Trebouxia* sp. S02/*Trebouxia* sp. 3A) being climatically the most distinct (Figure 5A).

Indeed, this cluster clearly differed by preferring cold-temperate, humid-in-summer environments (BIO5, 18, Wilcox tests *p* < 0.001 for all comparisons; Figures 5B,C), according to Rivas-Martínez et al. (2017) under non-Mediterranean bioclimates. The climatic differentiation of clusters 1, 4, and 7 was not significant (Wilcox tests, *p* < 0.05), but cluster 4 showed the least variance.

## Discussion

Although lichens are among the best examples of symbiotic associations, so far research has mainly been directed toward the fungal partner (mycobiont) and our knowledge about phycobiont diversity is still scarce. In fact, even in an ecologically relevant lichen genus, such as *Parmelia*, microalgae diversity is poorly known (Dumitru, 2019; Ossowska et al., 2019). The present study analyzed the diversity of *Trebouxia* associated with nine *Parmelia* spp., to arrive at a starting point regarding information about the myco-phycobiont interaction patterns, and provides the first insights into the ecological requirements of phycobionts associated with this genus, mainly in the Iberian Peninsula.

In general, lichen forming fungi associate with several phycobiont species belonging to a single microalgae genus. In *Parmelia*, the main phycobiont genus is *Trebouxia*. In this study, the genetic diversity of *Trebouxia* spp. was checked by using a nuclear region (nrITS barcode) and a chloroplast marker. In the case of the nrITS region, 15 *Trebouxia* microalgae species were detected in 159 specimens examined. According to the clade code mentioned for *Trebouxia*, all the sequences generated in this study belong exclusively to clade S and clade I. Chloroplast marker phylogeny corroborated the *Trebouxia* assignment at a clade level (S and I), but showed less resolution at a species level. These results agree with previous results (Ossowska et al., 2019) which pointed out a high diversity of *Trebouxia* (*Trebouxia* sp. S02, *Trebouxia* sp. S04, *Trebouxia* sp. S05, *Trebouxia* sp. Sun1, *Trebouxia* sp. Sun2) associated with three *Parmelia* spp. Some lineages are reported for the first time (*Trebouxia* aff. *flava*, *Trebouxia* sp. 1, *Trebouxia* sp. 2, *Trebouxia* sp. 3A and B, *Trebouxia* sp. 4A and B), while other already-known ones (*Trebouxia* sp. I02, *T. flava*, *Trebouxia* sp. S08, *T. suecica*, *Trebouxia* sp. S03, T. *australis*, and *T. angustilobata*) had never been found associated with *Parmelia* (only *Trebouxia* sp. S02 were previously described in *Parmelia*). The most frequent lineages of *Trebouxia* in the *Parmelia* spp. analyzed were *Trebouxia* sp. I02 and the new *Trebouxia* sp. 1. Previous studies had already found *Trebouxia* sp. I02 phycobionts in other genera, such as *Oropogon*, *Melanelixia*, *Melanohalea*, *Rhizoplaca*, and *Montanelia* (Leavitt et al., 2015). Other common lineages found are *T. flava* and *T. suecica*. For them, the range of associated host species has widened. Even though several authors focus their studies on the phycobiont distribution ranges (Blaha et al., 2006; Leavitt et al., 2016), our knowledge in this area is still incomplete. Our study helped us to expand the information concerning the distribution of different *Trebouxia* species. For example, *Trebouxia* sp. 3B or 4B were only found in Scotland and Spain, respectively. In contrast, *Trebouxia* sp. I02 were distributed among Spain, Romania, Norway, Ireland, the United States, and Canada. The richness in species of *Parmelia* added to the contrasting patterns of worldwide distributions in some *Parmelia* spp., and geographically restricted distributions in other species of this genus, could favor the high *Trebouxia* diversity found in this genus.

Climatic conditions are considered one of the most decisive factors influencing range species distribution (Currie et al., 2004). In fact, several authors have proven climatic variables to be good predictors of phycobiont diversity (Nelsen and Gargas, 2009; Fernández-Mendoza et al., 2011; Leavitt et al., 2016; Magain et al., 2017). In the present study, the distribution of both phycobionts and mycobionts of *Parmelia* lichens was almost completely explained by the symbiotic partner, climatic conditions and habitat (geography), which is really unexceptional. Climate explains part of the genetic variation of the phycobionts associated with *Parmelia*; however, most of the variation explained by climate is shared with other factors (Figure 4 and Supplementary Figure 4). These results suggest that the biodiversity patterns of the photobionts associated with the lichen forming genus *Parmelia* are multifactorial, with a mixture of factors acting together, as found in other genera (Vančurová et al., 2018; Jüriado et al., 2019). The identity of the partnership was clearly the main factor determining photobiont genetic variation, which agrees with the results found in many other lichens (Leavitt et al., 2015; Vančurová et al., 2018; Jüriado et al., 2019). This is expected in lichens with dominant asexual reproduction, such as the *Parmelia* spp. included in this study (Fernández-Mendoza et al., 2011; Steinová et al., 2019); the *Parmelia sulcata* group includes sorediate species, and the *Parmelia saxatilis* group mainly isidiate species, and both isidia and soredia are dispersal packages of both fungi and algae.

In the present work, mycobionts were studied by carrying out phylogenetic analyses of the nrITS region. This analysis was key to corroborate the accurate species identification and to segregate cryptic species. We estimated the delimitation of 13 species clusters. Ten of the lineages were placed in formally described species, whereas *P. serrana* B (which included samples from Sweden and Poland), *P. serrana* C and *P. saxatilis* C (from Spain) are undescribed lineages in *Parmelia*, and they are probably new cryptic species. The recent description of *P. rojoi* (= *P. saxatilis* B) in the Iberian Peninsula, and the fact that all species of PSAXgp distributed in Europe also occur in Spain, allowed Crespo et al. (2020) to hypothesized this area as a refugium during Pleistocene glaciations. The discovery of the new lineages (*P. serrana* C and *P. saxatilis* C) corroborates this hypothesis. *Parmelia serrana* and *Parmelia saxatilis* in PSAXgp are the most widespread taxon, and the most successful in colonizing diverse habitats in the Iberian Peninsula. Crespo et al. (2020) hypothesized that “the appearance of a Mediterranean climatic rhythm played a crucial role in the diversification of *P. serrana* in the Iberian Peninsula,” which could also have affected *P. saxatilis*. The presence of cryptic species inside *P. serrana* and *P. saxatilis* should be revised.

Finally, patterns in specificity and selectivity at a species level were analyzed for the 13 species of *Parmelia* identified. The mycobiont genus *Parmelia* varied its specificity toward the phycobiont depending on the level (group or species). Surprisingly, high specificity of each *Parmelia* group for a given *Trebouxia* clade level was observed: PSULgp associated only with clade I and PSAXgp with clade S. Differences in the presence of vegetative structures between both groups were previously highlighted (PSAXgp-isidiate vs. PSULgp-sorediate), thus supporting the idea proposed by Steinová et al. (2019) that ‘in *Cladonia* the specificity of the mycobiont toward the photobiont could be attributed to differences in the size of the vegetative propagules and the amount of them’. However, the degree of specificity is different within each group, since the PSAXgp mycobionts were less specific and associate with 12 *Trebouxia* spp., meanwhile those of PSULgp interacted only with three *Trebouxia* spp. In detail, as described by Smith et al. (2009), isidia contain both microalgae and fungal hyphae, and this cortex provide a tough outer surface. On other hand, soredium are produced from exposed medulla tissue and they are not enclosed in this protective outer cortical layer. These differences between isidia and soredia may influence phycobiont choice as, for example, sun irradiance, humidity and light exposure will be different in both structures. This very marked specificity of isidiates for clade S of *Trebouxia* and sorediate for clade I was probably already established when the divergence between isidiate and sorediate *Parmelia* occurred. Moreover, the new lineages, which have diversified, maintained the specificity for their respective clade.

Eight *Parmelia* species included in this study associate with only one *Trebouxia* lineage and could be considered specialist species. However, in some of these species, such as *P. mayi* and *P. serrana* C, a low number of specimens were sampled, thus making it difficult to establish if they are specialists or not. Five of the species studied cooperate with more than one *Trebouxia* (Figure 4); ranging from 2 to 5. The most generalist species are *P. saxatilis* A and *P. serrana* A, which associate with 5 and 4 *Trebouxia* spp., respectively. Several studies have reported the capability of mycobionts to associate with several phycobiont species instead of being specific toward one single microalgae lineage (Otálora et al., 2010). This is commonly considered as an adaptive strategy of the mycobiont to colonize a wider range of environmental conditions; selecting the best adapted phycobiont (Yahr et al., 2004, 2006; Casano et al., 2011; Muggia et al., 2013). In the case of lichen with asexual reproduction, association with more than one *Trebouxia* spp. could be related to the fact that the joint dispersal of fungal and algal does not always imply the maintenance of the same species of microalgae, but that once established the mycobiont can associate with other species of phycobionts (Dal Grande et al., 2018).

In general, it has been shown that species with worldwide distributions associate with a wide phycobiont range (Blaha et al., 2006; Muggia et al., 2014; Moya et al., 2017), while species growing in restricted areas are more specific (Otálora et al., 2010, 2013; Leavitt et al., 2016; Magain et al., 2017). However, there are exceptions to this rule, such as gypsum lichen species which colonized very restricted areas, but showed high flexibility in their phycobiont choice (Moya et al., 2021). Some species of *Parmelia* included in this study have broad geographical and ecological distribution ranges. Therefore, in these species (*P. sulcata* and *P. saxatilis*) the association with several *Trebouxia* species was expected, while for *P. encryptata*, with a more restricted distribution, lower diversity was to be assumed. However, since widely distributed *P. sulcata* associate with only one *Trebouxia* lineage, the differences in distribution ranges do not completely explain the differences found in specificity and selectivity patterns.

Moreover, colonization strategy and thallus morphology were described as key parameters in phycobiont choice and the range of associated phycobionts (Piercey-Normore, 2004; Bačkor et al., 2010; Moya et al., 2020). Piercey-Normore (2004) pointed out these factors to explain the association myco/phycobiont patterns in *Cladonia*. More recently, Moya et al. (2017, 2021) and Molins et al. (2021) underlined that different thallus parts of crustose or fruticulose lichen species could harbor different microalgal compositions. Also, Leavitt et al. (2015) highlighted evidence of the correlation between thalli growth forms and selectivity in Parmeliaceae mycobionts. In the present study, all the species included show similar substrata i.e., rocks and barks (although PSULgp spp. are epiphytes) and foliaceous patterns. Summarizing, our results show that interaction patterns in *Parmelia* may be influenced by a mixture of factors, including the type of vegetative structure, mycobiont distribution and habitat, with Cluster 6 (*P. saxatilis* A/*P. saxatilis* B/*Trebouxia* sp. 2/*Trebouxia* sp. S02/*Trebouxia* sp. 3A) being climatically the most distinct by preferring cold-temperate, humid-in-summer environments (non- Mediterranean bioclimates).

## Data Availability Statement

The data presented in the study are deposited in the GenBank repository, and the accession number is provided in Supplementary Table 1.

## Author Contributions

PM, AM, SC, and EB conceptualized the study. AM, SC, and CD performed the experiments and generated the data. PM, AM, PŠ, and PD performed by the formal analysis. EB, AC, MM, PŠ, and PD provided resources, funding, and supervision. EB provided project administration. All authors contributed to writing, reviewing and editing.

## Conflict of Interest

The authors declare that the research was conducted in the absence of any commercial or financial relationships that could be construed as a potential conflict of interest.

## Publisher’s Note

All claims expressed in this article are solely those of the authors and do not necessarily represent those of their affiliated organizations, or those of the publisher, the editors and the reviewers. Any product that may be evaluated in this article, or claim that may be made by its manufacturer, is not guaranteed or endorsed by the publisher.

## Footnotes

1
http://technelysium.com.au/wp/chromas/

2
http://beast.bio.ed.ac.uk

3
http://species.h-its.org/ptp/

4
https://bioinfo.mnhn.fr/abi/public/abgd/abgdweb.html

## Supplementary Material

Supplementary Figure 1 Summary of the three species delimitation analyses (GMYC, bPTP, and ABGD) using mycobiont nrITS sequences. The topology shown corresponds to the Bayesian ultrametric tree obtained in BEAST. Each color corresponds to the different fungal species delimited.

Supplementary Figure 2 *Trebouxia* phylogenetic analysis. Rooted LSU rDNA gene tree representing 139 *Trebouxia* sequences, including 9 well−accepted *Trebouxia* species from SAG and UTEX retrieved from the GenBank. Newly generated sequences are marked as ex *Parmelia* spp._locality-code number. Seven *Trebouxia* species detected were indicated. Values at nodes indicate statistical support estimated by two methods: bootstrap support (BS, RAxML analysis) and posterior probabilities (PP, MrBayes analysis). Scale bar shows the estimated number of substitutions per site.

Supplementary Figure 3 Summary of the three species delimitation analyses (GMYC, bPTP, and ABGD) using concatenated phycobiont nrITS and LSU DNA dataset. The topology shown corresponds to the Bayesian ultrametric tree obtained in BEAST. Each color corresponds to the different algal species delimited.

Supplementary Figure 4 **(A)** Network diagram showing the location of the seven clusters (1–7) **(B)** Venn’s diagram showing the variation in distribution of the clusters explained by effects of climate, geography and substrate.

Supplementary Figure 5 PCA result of seven clusters distribution depending on climatic factors. Large circles represent group centroids. Climatic data were obtained from the Global Climate Data—WorldClim. BIO1, Annual Mean Temperature; BIO2, Mean Diurnal Range [Mean of monthly (max temp—min temp)]; BIO3, Isothermality (BIO2/BIO7) (× 100); BIO4, Temperature Seasonality (standard deviation × 100); BIO5, Max Temperature of Warmest Month; BIO6, Min Temperature of Coldest Month; BIO7, Temperature Annual Range (BIO5-BIO6); BIO8, Mean Temperature of Wettest Quarter; BIO9, Mean Temperature of Driest Quarter; BIO10, Mean Temperature of Warmest Quarter; BIO11, Mean Temperature of Coldest Quarter; BIO12, Annual Precipitation; BIO13, Precipitation of Wettest Month; BIO14, Precipitation of Driest Month; BIO15, Precipitation Seasonality (Coefficient of Variation); BIO16, Precipitation of Wettest Quarter; BIO17, Precipitation of Driest Quarter; BIO18, Precipitation of Warmest Quarter; BIO19, Precipitation of Coldest Quarter.

Supplementary Table 1 Samples used in this study, with details on collection data, herbarium codes and the GenBank accession numbers for the sequences generated for the nrITS (mycobiont and microalgae) and LSU rDNA.
