# Supplementary figures and images for: Biodiversity Patterns and Ecological Preferences of the Photobionts Associated With the Lichen-Forming Genus Parmelia
Source: Front Microbiol. 2021 Dec 24;12:765310. doi: 10.3389/fmicb.2021.765310 (PMC8739953; doi:10.3389/fmicb.2021.765310)

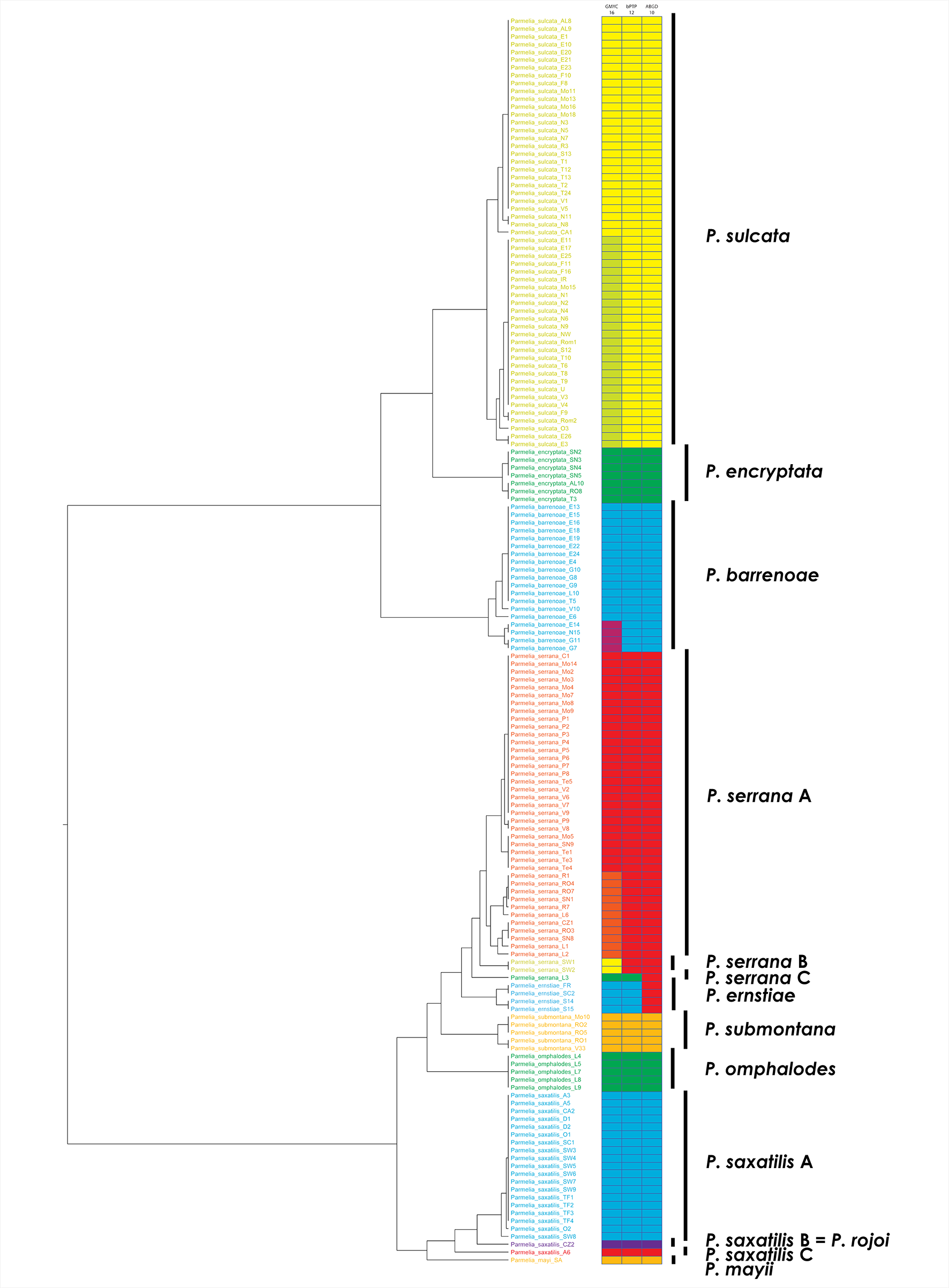

Supplement: Supplementary Figure 1 — Summary of the three species delimitation analyses (GMYC, bPTP, and ABGD) using mycobiont nrITS sequences. The topology shown corresponds to the Bayesian ultrametric tree obtained in BEAST. Each color corresponds to the different fungal species delimited. [file Image_1.TIF]

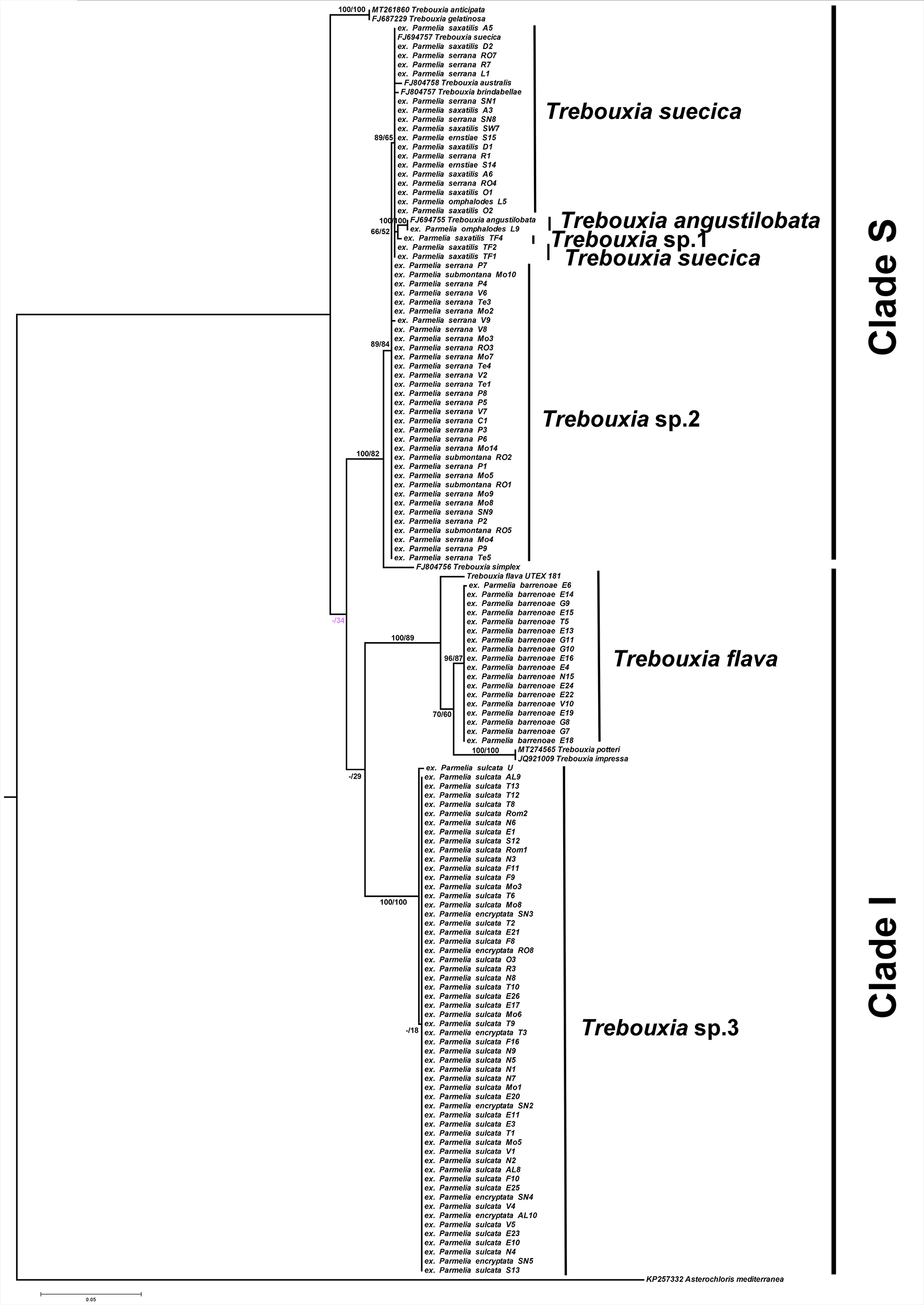

Supplement: Supplementary Figure 2 — Trebouxia phylogenetic analysis. Rooted LSU rDNA gene tree representing 139 Trebouxia sequences, including 9 well−accepted Trebouxia species from SAG and UTEX retrieved from the GenBank. Newly generated sequences are marked as ex Parmelia spp._locality-code number. Seven Trebouxia species detected were indicated. Values at nodes indicate statistical support estimated by two methods: bootstrap support (BS, RAxML analysis) and posterior probabilities (PP, MrBayes analysis). Scale bar shows the estimated number of substitutions per site. [file Image_2.TIF]

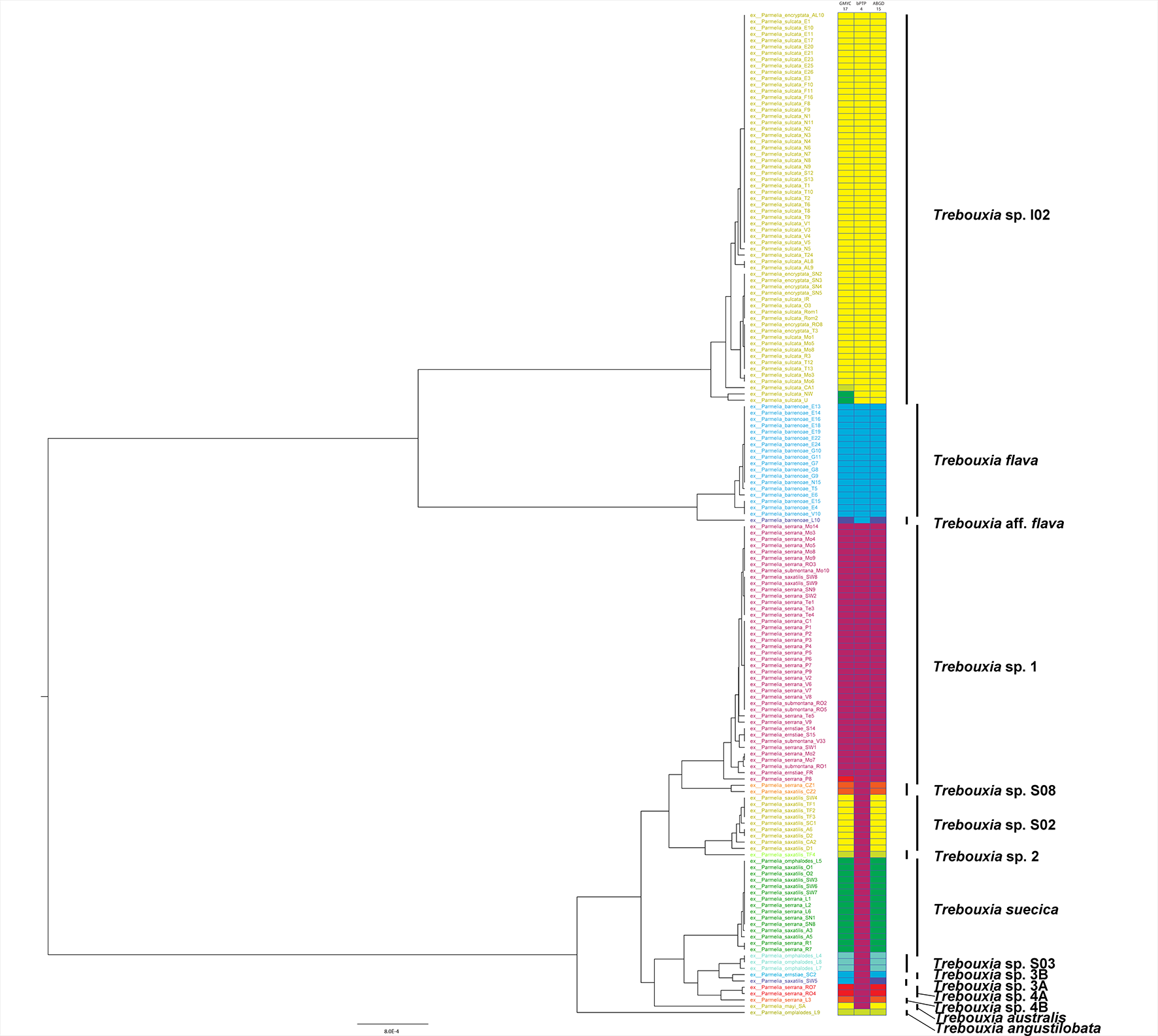

Supplement: Supplementary Figure 3 — Summary of the three species delimitation analyses (GMYC, bPTP, and ABGD) using concatenated phycobiont nrITS and LSU DNA dataset. The topology shown corresponds to the Bayesian ultrametric tree obtained in BEAST. Each color corresponds to the different algal species delimited. [file Image_3.TIF]

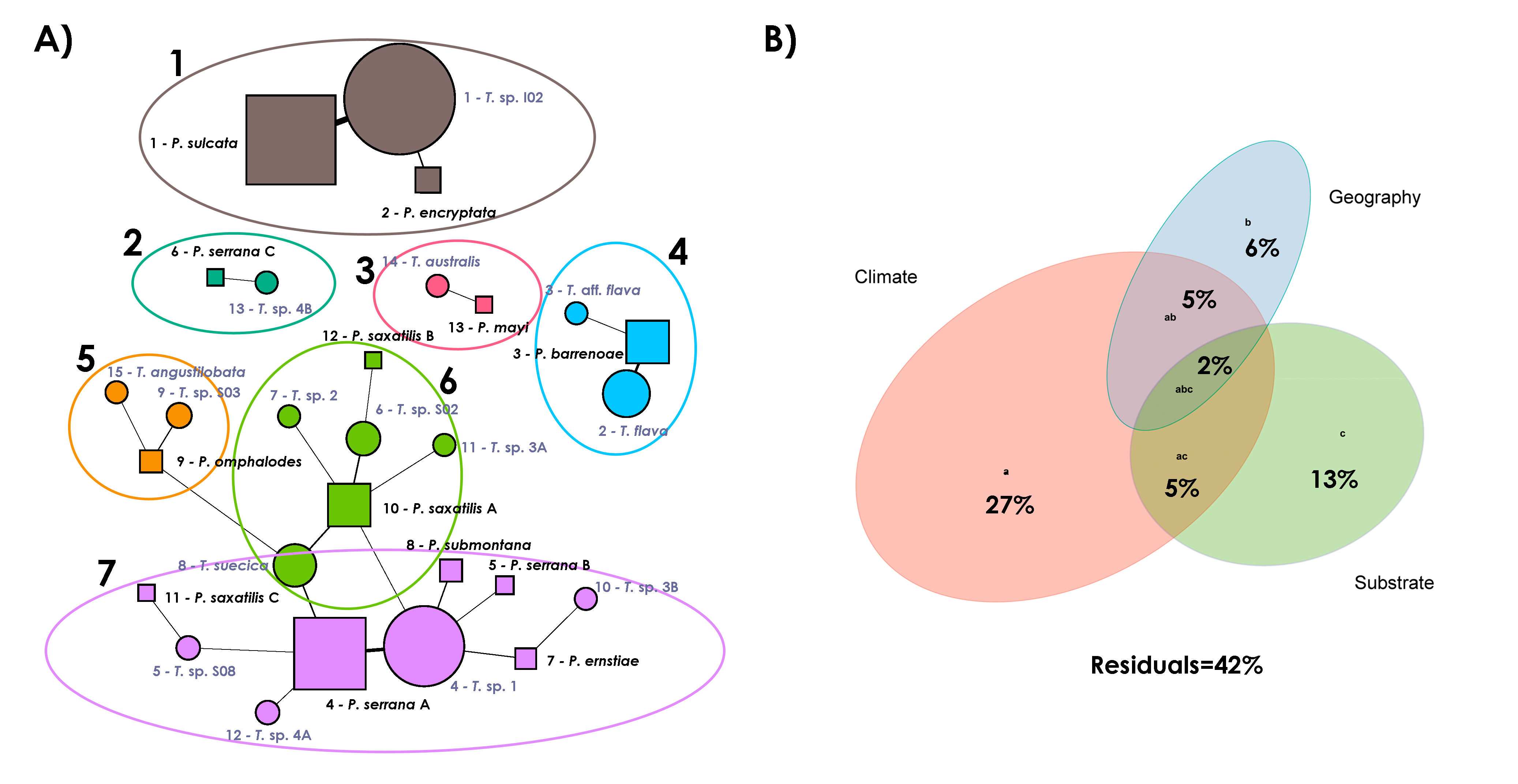

Supplement: Supplementary Figure 4 — (A) Network diagram showing the location of the seven clusters (1–7) (B) Venn’s diagram showing the variation in distribution of the clusters explained by effects of climate, geography and substrate. [file Image_4.TIF]

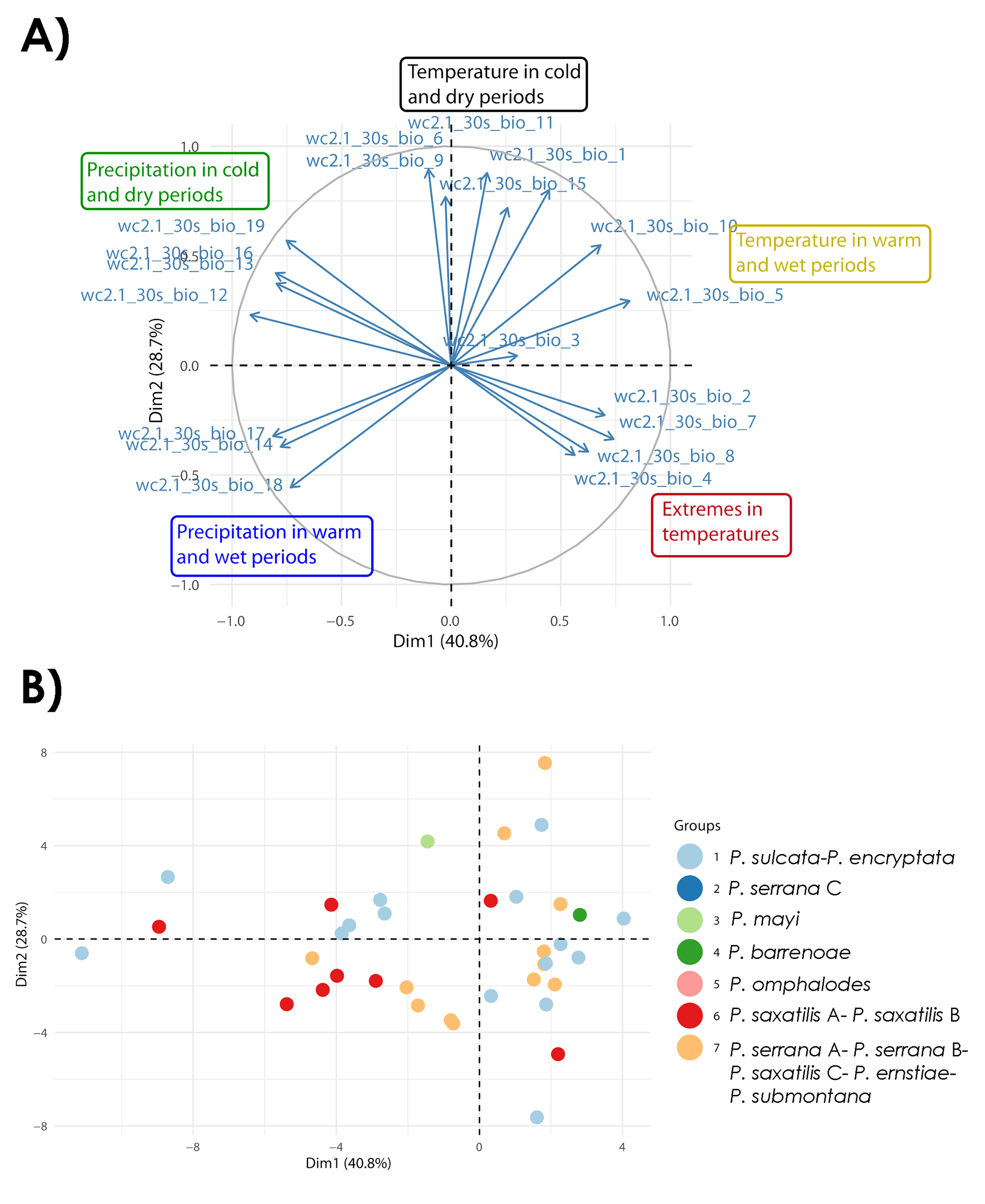

Supplement: Supplementary Figure 5 — PCA result of seven clusters distribution depending on climatic factors. Large circles represent group centroids. Climatic data were obtained from the Global Climate Data—WorldClim. BIO1, Annual Mean Temperature; BIO2, Mean Diurnal Range [Mean of monthly (max temp—min temp)]; BIO3, Isothermality (BIO2/BIO7) (× 100); BIO4, Temperature Seasonality (standard deviation × 100); BIO5, Max Temperature of Warmest Month; BIO6, Min Temperature of Coldest Month; BIO7, Temperature Annual Range (BIO5-BIO6); BIO8, Mean Temperature of Wettest Quarter; BIO9, Mean Temperature of Driest Quarter; BIO10, Mean Temperature of Warmest Quarter; BIO11, Mean Temperature of Coldest Quarter; BIO12, Annual Precipitation; BIO13, Precipitation of Wettest Month; BIO14, Precipitation of Driest Month; BIO15, Precipitation Seasonality (Coefficient of Variation); BIO16, Precipitation of Wettest Quarter; BIO17, Precipitation of Driest Quarter; BIO18, Precipitation of Warmest Quarter; BIO19, Precipitation of Coldest Quarter. [file Image_5.TIF]
